# Subchondral bone microenvironment in osteoarthritis and pain

**DOI:** 10.1038/s41413-021-00147-z

**Published:** 2021-03-17

**Authors:** Yan Hu, Xiao Chen, Sicheng Wang, Yingying Jing, Jiacan Su

**Affiliations:** 1 Department of Orthopedics Trauma, Shanghai Changhai Hospital, Naval Military Medical University, Shanghai, China; 2 Institute of Translational Medicine, Shanghai University, Shanghai, China; 3 Department of Orthopedics, Shanghai Zhongye Hospital, Shanghai, China

**Keywords:** Calcium and phosphate metabolic disorders, Metabolic disorders

## Abstract

Osteoarthritis comprises several joint disorders characterized by articular cartilage degeneration and persistent pain, causing disability and economic burden. The incidence of osteoarthritis is rapidly increasing worldwide due to aging and obesity trends. Basic and clinical research on osteoarthritis has been carried out for decades, but many questions remain unanswered. The exact role of subchondral bone during the initiation and progression osteoarthritis remains unclear. Accumulating evidence shows that subchondral bone lesions, including bone marrow edema and angiogenesis, develop earlier than cartilage degeneration. Clinical interventions targeting subchondral bone have shown therapeutic potential, while others targeting cartilage have yielded disappointing results. Abnormal subchondral bone remodeling, angiogenesis and sensory nerve innervation contribute directly or indirectly to cartilage destruction and pain. This review is about bone-cartilage crosstalk, the subchondral microenvironment and the critical role of both in osteoarthritis progression. It also provides an update on the pathogenesis of and interventions for osteoarthritis and future research targeting subchondral bone.

## Introduction

Osteoarthritis (OA) is a series of diseases characterized by articular cartilage destruction and persistent pain.^1^ The prevalence of OA is increasing, with an estimated number of patients of 250 million, and its risk factors include aging, sex, obesity, chronic systemic diseases, genetics, and injury. OA is believed to be a whole-joint disorder involving the bone, cartilage, synovium, ligament and joint capsule. The pathogenesis of OA involves at minimum mechanical factors, inflammation and metabolism, and these factors interact with each other, thus leading to poor curative effects for most treatments, except joint replacement at the end stage.

The initial pathological changes in OA have remained unclear until now, but it is certain that various pathologies associated with OA could worsen in combination, including cartilage degradation, subchondral bone sclerosis, angiogenesis and nerve innervation. Current therapies for symptomatic OA can be divided into several types: oral drug administration, intraarticular drug injection, intravenous drug administration and surgical operations. Generally, oral medications include NSAIDs and glucosamine; medicines injected into the joint space include sodium hyaluronate, glucocorticoids and anesthetics; and surgical options include but are not limited to total or partial joint replacement, arthroscopic surgery and high tibial osteotomy. Significantly, there is no single therapy that can completely resolve this disease with a complex origin, and further classification of OA subtypes based on their pathogenesis could be the only method of disease resolution in the future. As therapies aimed at articular cartilage or synovial components are not satisfactory and the mechanism of bone-cartilage crosstalk is gradually becoming clear, we reviewed the critical role of subchondral bone in osteoarthritis and concluded that subchondral bone could be an ideal “entry point” for osteoarthritis treatment.

## Subchondral bone microenvironment in OA

Subchondral bone refers to the bony layer beneath the hyaline cartilage and cement line and can be divided into two parts anatomically, the subchondral bone plate (SBP) and subchondral bone trabecula. The SBP is a compact, polyporous calcified plate crisscrossed by multiple vessels and nerve fibers. Subchondral bone trabeculae are cancellous bony structures subjacent to the SBP that undergo continuous bone remodeling. Subchondral bone provides both mechanical and nutritional support for cartilage, indicating that microenvironmental changes in subchondral bone might affect cartilage metabolism, directly or indirectly.

### Subchondral bone marrow lesions

A bone marrow lesion (BML), or so-called “bone marrow edema”^2^ or “bone marrow edema-like abnormality”,^3^ describes a noncystic, ill-defined, high-signal area on T2-weighted or proton density-weighted MRI.^4^ BMLs are divided into the subchondral type and ligamentous type,^5^ depending on the location. Subchondral BMLs (SBMLs), which result in abnormal signals on MRI beneath the calcified cartilage layer, affect more than half of asymptomatic individuals over 50, and their prevalence increases with age.^6^ SBMLs can be observed in the early stage of knee OA and are thought to be helpful in early screening.

Interestingly, in approximately two-thirds of OA patients with SBMLs, cartilage erosion occurs around the superficial area of the SBMLs,^5^ indicating the underlying linkage between SBMLs and OA progression. The worsening of SBMLs based on MRI manifestations is associated with subsequent radiographic findings and persistent pain.^7–9^ At the site of an SBML, a high in situ turnover rate, pain sensitization and proinflammatory signaling activation have been observed through histological analysis and microarray techniques,^10^ finally resulting in increased bone mineral density (BMD) and subchondral sclerosis.^11^ Clinical observations have shown that over a period of 24 months, the size of the subchondral high-signal area on MRI increases, particularly in the medial tibia plateau and medial condyle, presenting a strong relationship with the loss of cartilage volume in corresponding regions.^12^ Recent clinical data have shown the strong association between BMLs and cartilage damage in the tibial plateau, indicating the diagnostic value of predicting the degenerative status within the osteochondral unit.^13^

### Subchondral bone cells around the chondro-osseous junction

Mesenchymal stromal cells (MSCs) are cell clusters with the capacity for self-renewal and the ability to differentiate into adipocytes, osteoblasts, chondrocytes, and myocytes in an appropriate microenvironment. During an in vivo experiment, transforming growth factor (TGF)-β3-induced MSC homing completely repaired the cartilage structure without xenogeneic cell implantation,^14^ indicating that a robust subchondral bone microenvironment might support full-thickness articular cartilage regeneration. Evidence suggests that enriched MSCs derived from synovium, synovial fluid^15^ or periarticular adipose tissue^16^ also participate in cartilage regeneration in multiple pathological conditions, including OA.^17^ Despite the participation of MSCs of multiple origins, the presence of spontaneous cartilage formation in full-thickness defects in vivo leads to better consequences,^18^ suggesting that an intact subchondral bone environment might contribute to the self-repair process more than the MSC population.

As matrix resorption cells, osteoclasts act as “conductors” in subchondral bone metabolism and are involved in not only bone resorption but also type H vessel location and sensory nerve innervation. Tartrate-resistant acid phosphatase-positive (TRAP^+^) cells located around the chondro-osseous junction area exhibit lower cathepsin K (Ctsk) expression and fewer nuclei than those in the marrow space.^19^ Erosion of the growth plate by endothelial cells during bone development mediates bone elongation, and the Ctsk^low^ osteoclast subtype plays a critical role in inducing the side-to-side anastomosis of blind-ended vessels. These so-called “vessel-associated osteoclasts” show a high affinity for type H vessels, which is supported by the receptor activator of nuclear factor-κB ligand (RANKL) expression of endothelial cells and induces the anastomosis of type H vessels.

### Bone-cartilage interaction

The tidemark, or hyaline-calcified cartilage interface, was first reported in 1984 and has been found to shift slowly toward the joint space during aging.^20^ Material exchanges across the tidemark or cement line are critical for cartilage metabolism in the absence of capillaries inside hyaline cartilage. The chondrocyte extracellular matrix comprises nearly 70%–80% water, collagen and proteoglycans, and it is impermeable to anionic molecules owing to the negative potential of proteoglycans. Because of this characteristic and the increase in the proteoglycan concentration with depth, an anionic contrast agent creates a density gradient in the cartilage layer,^21^ so contrast-enhanced CT could help to diagnose early OA since anionic media uptake is accelerated at sites of cartilage injury.^22^ Cationic contrast agents also have potential in the diagnosis of cartilage damage on imaging based on the electrostatic attraction effect; using nonionic agents, the collagen concentration can be mapped on contrast-enhanced CT.^23^

Evidence has demonstrated the particular relationship of mutual regulation between cartilage and subchondral bone. Experiments on small molecular diffusion have revealed the existence of direct molecular signaling linking cartilage and subchondral bone,^24^ and this crosstalk could increase in OA, suggesting that the subchondral bone microenvironment is involved in cartilage degeneration in OA. The inhibition of TFG-β in subchondral bone MSCs results in a protective effect on chondrocytes, indicating that TGF-β-induced osteogenesis plays an important role in bone-cartilage crosstalk.^25^ Chondrocytes could participate in subchondral remodeling through the RANKL/RANK/OPG signaling pathway, and RANKL and OPG produced by chondrocytes could regulate bone metastasis in certain situations, but their specific roles remain unknown.^26^ During the epiphyseal extension process, chondrocyte-derived OPG facilitates bone formation by blocking osteoclast maturation^27,28^ through β-catenin signaling,^29^ and the senescence-associated secretory phenotype (SASP), which is characterized by the secretion of chemokines and proinflammatory cytokines, accelerates OA progression.^30^ Hypertrophic chondrocyte-derived vascular endothelial growth factor (VEGF) participates in the recruitment of MSCs and endotheliocytes, thus promoting bone formation, vessel innervation and cartilage resorption.^31^

Direct cell-to-cell contact has been observed in some pathological sections,^32^ in addition to indirect molecular crosstalk. Interestingly, the tidemark is not a single line but a complex three-dimensional structure. CT has revealed that the cartilage layer penetrates the calcified layer and eventually reaches the subchondral bone plate in OA,^33^ further indicating direct cell-to-cell communication among chondrocytes, osteocytes, and osteoclasts. In an ex vivo coculture system, MSCs promoted matrix production and chondrocyte proliferation through a trophic-associated mechanism instead of chondrogenic differentiation.^34,35^ Chemotactic factors released from MSCs recruit endogenous stem cells to cartilage damage sites and facilitate regeneration,^36^ and exosomes derived from MSCs have demonstrated analogous effects in multiple in vivo experiments.^37–40^ Moreover, subchondral bone osteoblasts derived from the zone of sclerosis in OA patients altered the chondrocyte phenotype in vitro and accelerated hypertrophic differentiation and calcification.^41^ Taken together, these clues suggest that cartilage and subchondral bone regulate each other reciprocally through both cellular and molecular signaling.

### Uncoupling of bone remodeling in the OA subchondral bone microenvironment

#### High turnover rate

**Fig. 1:**
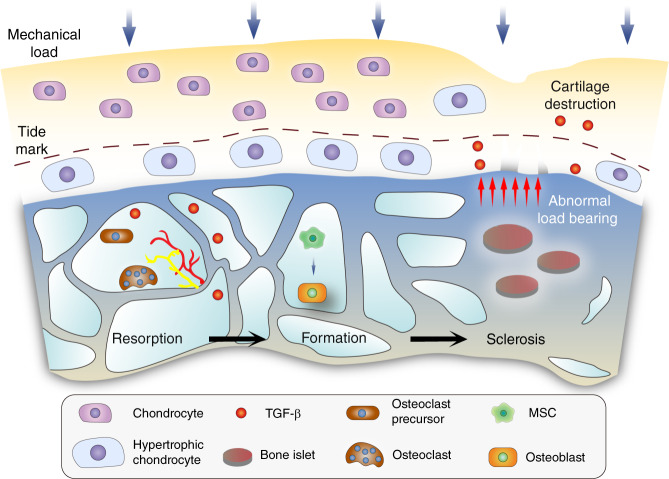
Schematic diagram of subchondral bone responding to aberrant mechanical loading and subsequent cartilage destruction. In the early stage of knee OA, bone resorption is overactivated, especially in the BML area. Biological factors, such as TGF-β, orchestrate MSC recruitment and bone formation, along with the generation of vessels and nerve fibers. Osteopetrosis and bone islets can be observed in advanced stages, which induce cartilage degeneration, at least in part, by abnormal load bearing

Relatively balanced modeling and remodeling processes maintain the subchondral bone microenvironment.^42^ Once the mechanical load changes or any other uncertain etiology occurs, the turnover rate of subchondral bone adjusts to adapt (Fig. 1). An elevated osteoclast number has been observed in samples from both humans and mice with OA, accompanied by aberrant subchondral bony structures and mechanical support for the superficial layer. Cumulative evidence has shown that osteocytes and hypertrophic chondrocytes, rather than osteoblastic cells, serve as major suppliers of RANKL during the remodeling process.^28,43^ Since osteocytes are a critical component in identification of the skeletal load,^44^ abnormal mechanical force activates RANKL signaling and osteoclastogenesis, leading to aberrant bone remodeling and osteosclerosis. In addition, *Dmp1*-Cre; *RANKL* ^f/f^ mice presented a significant increase in osteoclasts in a tail-suspension experiment, demonstrating the role of chondroblastic RANKL signaling in osteoclastogenesis. Subsequent evidence has shown that RANKL expression and production are elevated in arthritis models.^26,45^ Furthermore, soluble RANKL has a sufficiently small molecular weight to pass through subchondral bone plate cavities,^28^ thus allowing it to play a role in osteoclast maturation.^46^

Bone marrow lesions were found in femoral head samples obtained from patients undergoing total hip arthroplasty, along with a high bone formation rate, hyperplastic woven bone and excessive angiogenesis.^47^ Furthermore, a significant association between the subchondral bone turnover rate and cartilage damage has been proven by (99m)Tc-SPECT and 3-T MRI.^48^ An elevated number and distribution of osteoclasts adjacent to the trabeculae are representative phenomena in SBMLs.^49^ Subsequent remodeling processes are associated with angiogenesis and nerve growth, and the inhibition of osteoclast activity, angiogenesis or even nerve growth in subchondral bone marrow lesion sites might have potential therapeutic effects against both OA progression and pain. Siebelt et al. revealed that the subcutaneous injection of alendronate reduces both bone turnover and osteoarthritis progression.^50^ The preemptive administration of alendronate before OA induction has been found to prevent subchondral bone turnover, cartilage degeneration and osteoarthritis pain better than early or delayed treatment after model establishment.^51^ Taken together, these findings indicate that in OA, subchondral bone undergoes an aberrant remodeling process with an elevated turnover rate under the regulation of both mechanical and biological signals.

#### Microstructural changes

**Fig. 2:**
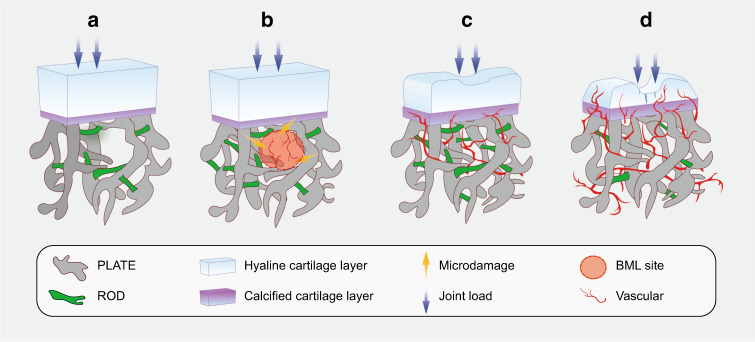
Subchondral bone microstructural transformation during OA progression. Rod-like and plate-like trabeculae are distributed accurately at a proper ratio to disperse stress. Osteogenesis coupled with angiogenesis lead to an increased BMD and conspicuous microstructural changes at the subchondral level. A decrease in the rod/plate ratio and subchondral bone plate thickening result in excessive mechanical support, which is detrimental to chondrocyte metabolism. Vessel-induced digestion of the cartilage matrix from the bottom up also takes part in the progression of cartilage destruction. **a** Normal state; **b** early stage with BML and vascularization; **c** progressive stage with cartilage damage; **d** end stage with severe cartilage destruction and vessel erosion

Beneath the hyaline and calcified cartilage, subchondral bone undergoes precise modeling processes to maintain a normal microstructure.^52^ According to Wolff’s Law, the internal architecture of bone develops according to the magnitude and direction of the applied load.^53^ Mounting evidence has indicated that microstructural changes occur during the course of OA, as determined by morphometric assessment methods^54^ and shown in Fig. 2. Compared with uninjured joints, more pronounced subchondral bone lesions are observed in joints with meniscus tears or ligament damage,^55^ suggesting that microstructural changes might be considered a contributor to secondary disease caused by alignment issues. The subchondral bone plate changes prior to trabeculae in OA and restricts bone-cartilage substance interchange via its internal properties, such as thickness and porosity.^56^ Living osteocytes show persistent metabolic activity, including perilacunar/canalicular remodeling (PLR).^57^ Osteocytes digest and reestablish the matrix around them through the secretion of matrix metalloproteinase (MMP),^58^ cathepsin K (ctsK),^59^ carbonic anhydrase 2 (CA2)^60^ and other enzymes. Interestingly, the elimination of MMP-13 in osteocytes while maintaining its expression in chondrocytes degrades the canalicular structure and accelerates degradation in OA. A comparison between a cadaveric control and subchondral bone with OA supports this conclusion, indicating an association between subchondral bone microstructure and OA progression.^61^ Subchondral bone trabeculae include rod-like and plate-like structures according to their spatial morphology.^62^ A quantitative CT analysis of human samples revealed a higher bone volume, thicker plate and lower trabecular rod/plate ratio on the medial plateau than on the lateral side.^63^ In addition, excessive pressure in obesity-related osteoarthritis leads to lateral deformation of the articular cartilage and creates horizontal fissuring at the osteochondral interface,^64^ based on the observation of human samples. Analogous subchondral bone microstructural changes occur in the patella during knee osteoarthritis,^65^ as well as the talus in ankle osteoarthritis. Diabetes mellitus has been shown to be a risk factor for osteoarthritis,^66^ and significant mechanical impairments are observed even in patients without OA pain or cartilage attenuation,^67^ supporting the idea that subchondral bone microstructural damage might be an important predictor of OA progression.

#### TGF-β signaling

TGF-β1-3 is a functional growth factor secreted by multiple cells that are present only in mammals. The TGF-β superfamily participates in cell proliferation, differentiation, migration and apoptosis by sensing biological clues and mediating their signaling to serve as bioactive sentries.^68^ TGF-β acts as an important growth factor regulating cell migration, the epithelial-mesenchymal transition and other biological functions, and global defects in TGF-β lead to various major diseases, such as organ fibrosis and musculoskeletal disorders, including cartilage destruction. After secretion, TGF-β is normally stored in the extracellular matrix in a latent form,^69^ or with so-called latency-associated peptide (LAP), and it can be cleaved into the mature form and latency-associated peptide in the proper environment. This inactive form enables the spatial and temporary activation of TGF-β for diverse physiological needs. Several TGF-β activators have been identified to date, most of which are markers of extracellular matrix (ECM) disorders, such as MMP,^70^ plasmin,^71^ and thrombospondin-1 (TSP1),^72^ as well as an appropriate pH.^73^ TGF-β1 is abundant in the bone matrix, cartilage and skin, where it regulates the physical activities of these organs or tissues.^74^ During bone resorption, a mildly acidic environment is generated around the resorption site at a pH of 4.5, releasing mature TGF-β1 and thus launching remodeling and modeling processes.^75^

Activated TGF-β binds specific receptors, named receptor I and receptor II; subsequent signaling is dependent on their complex forms and occurs mainly via Smad-mediated or Smad-independent pathways.^68^ Stockpiles released from the bone matrix orchestrate MSC migration and differentiation and repair of the resorptive space. Specifically, TGF-β1 recruits MSCs in a gradient-dependent manner,^76^ while other factors, such as IGF-1,^77^ induce osteoblastogenesis. TGF-β also facilitates tissue repair by increasing MSC proliferation, mobilization and migration. The tissue regeneration process requires proper migration and accurate differentiation, and previous studies have reported that colonic crypt,^78^ hair follicle^79^ and renal fibrosis^80^ repair could be accelerated by TGF-β. In a rabbit humeral cartilage defect model, cell-free scaffolds carrying TGF-β3 recruited significantly more MSCs than the negative control and achieved full regeneration without exogenous stem cell implantation.^14^ In addition, TGF-β-regulating signaling also plays a role in the PLR by maintaining homeostasis of the osteocyte matrix.^81^

**Fig. 3:**
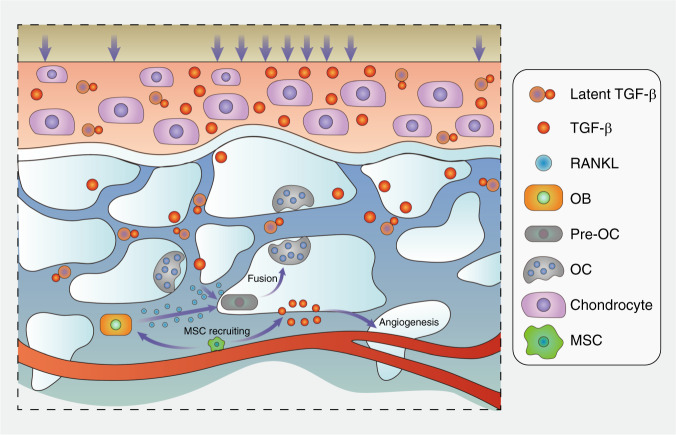
TGF-β signaling in the osteochondral area in OA. Shear force breaks the spatial structure of latent TGF-β, thus releasing activated TGF-β and accelerating bone remodeling. On the other hand, overactivated osteoclasts enable TGF-β expression, recruit MSCs and facilitate angiogenesis. In turn, bone and vessel formation promote cartilage TGF-β mobilization, and the cycle continues

Aberrant TGF-β activation and subsequent signal transduction lead to multiple disease phenotypes, such as osteoarthritis (Fig. 3), enthesopathy,^82^ and Camurati-Engelmann disease (CED). The mutation of TGF-β1 causes CED, which is characterized by thickened cortical bone and narrowing of the marrow space^83^ due to excessive TGF-β1 activation. A high incidence of osteoarthritis was observed among CED patients in former clinical research,^84^ along with elevated active TGF-β levels. Additionally, the inhibition of subchondral TGF-β delayed OA progression, and knockout of the TGF-β type II receptor on MSCs achieved a similar effect in vivo.^25^ Overall, subchondral osteocytes sense aberrant mechanical stress, induce osteoclast maturation through RANKL/RANK signaling,^43^ and release TGF-β and other bioactive factors via both osteoclast resorption and the PLR process. Subsequently, endogenic MSC migration and unexpected bone islet formation disrupt mechanical homeostasis below the cartilage, which promotes chondrocyte apoptosis and substrate degradation. Anomalous hyaline and calcified cartilage place exceptional pressure on the subchondral region, further amplifying the force-induced turnover rate and creating a positive feedback loop to accelerate OA progression. Interestingly, mechanical force application could break the latent formation of LAP and release activated TGF-β,^85^ which indicates that abnormal mechanical stress might play a role in cartilage TGF-β signaling activation, thus disturbing chondrocyte metabolism.

#### Hypervascularization

**Fig. 4:**
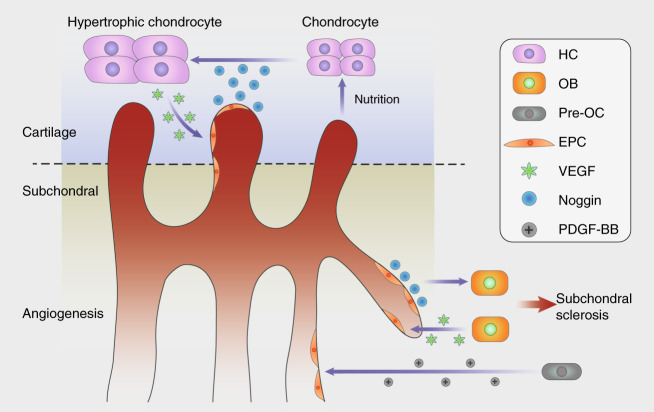
Coupled angiogenesis and osteogenesis in the subchondral bone accelerate cartilage destruction. Activated TGF-β and PDGF-BB derived from pre-OCs lead to neovascularization, type H vessels couple angiogenesis and osteogenesis by Notch signaling, and OBs accelerate this cycle via RANKL and VEGF release. Endothelial cells digest cartilage and contribute to chondrocyte hypertrophy, which in turn attracts vascular invasion by VEGF signaling

Osteogenesis and angiogenesis are precisely coupled via type H vessels and multiple cytokines in bone metabolism.^86^ Recent evidence has revealed a positive feedback loop in MSCs and type H vessels.^87^ Platelet-derived growth factor receptor (PDGFR)-positive mesenchymal cells are spatially consistent with type H vessels, and angiogenic endothelial cells recruit MSCs through chemotaxis and promote osteogenesis.^88^ Key signaling pathways in MSCs coupled with endothelial cells include the TGF-β,^89^ PDGF-PDGFR,^90^ angiopoietin,^91^ Notch,^92^ and FAK signaling pathways.^93^ Thus, blocking the interaction between aberrant osteogenesis and angiogenesis might have a therapeutic effect on excessive subchondral osteosclerosis (Fig. 4).

Hyaline cartilage is a nonvascularized tissue with resistance to vessel formation, but angiogenesis plays a critical role in endochondral bone formation,^94,95^ suggesting that vessels might be responsible for cartilage digestion. Elegant research presented by Ramasamy et al.^19^ proved the proteolytic function of endothelial cells, which misdirected the orientation of growth plate type H vessel elongation in the proximal tibia. Hypertrophic chondrocytes are a major source of VEGF, which links cartilage resorption, ossification and angiogenesis.^96^ VEGF derived from osteoblasts can recruit osteoclasts and stimulate osteogenesis and angiogenesis,^31^ and the effects of VEGF in the joint space can be blocked with an antagonist to hinder repair by repelling vascular invasion.^97,98^ For maintenance of the avascular environment, chondromodulin (Chm-1),^99^ TSP-1^100^ and troponin 1 (Tn1)^101^ have been shown to counter cartilage angiogenic factors, such as VEGF, fibroblast growth factor (FGF) and TGF-β.

Resistance to vascular invasion in the joint decays in OA, and the spatial orientation of protease inhibitors makes them unable to expel vessels around the tidemark. Duplication of the tidemark and the penetration of vessels and nerve fibers have been observed in clinical OA samples via both micro-CT and high-frequency ultrasound.^102^ Both angiogenic and antiangiogenic factors are overexpressed in OA chondrocytes, but protease inhibitors remain at normal levels in the deep layer of cartilage adjacent to the bony plate,^103^ which might allow bottom-up neovascular invasion. In fact, the number of cartilage-resorptive type H vessels has been shown to be increased in subchondral bone in mouse models and human patients. Activated OA chondrocyte-derived mammalian target of rapamycin complex 1 (mTOR1) signaling stimulates vessel invasion partially through the upregulation of VEGF-A expression, while nutrition optimization in capillaries in turn promotes mTORC1 activation.^104^

#### Sensory nerve innervation

Nerve growth is normally coupled with angiogenesis under both physiological and pathological circumstances,^105^ and perivascular cells stimulate nerve growth and help new axons navigate to their destination. Nerve fibers appear nearly two weeks after angiogenesis in rodent models.^106^ Nerve growth factor (NGF) has been proven to stimulate both vascular and nerve growth in subchondral bone in OA and thus link angiogenesis and pain in the joint in OA.^107^ Specifically, chondrocyte-derived NGF expression increases in the OA microenvironment and upregulates the expression of fibroblast growth factor in a dose- and time-dependent manner, thus promoting the angiogenic potential of endothelial cells.^108^ Nerve growth and maintenance are also related to pleiotropic factors, such as VEGF and TGF-β, which are highly expressed in osteochondral junction areas in OA. Four types of axon guidance molecules, including netrins, slits, ephrins and semaphorins, are responsible for directing fiber extension and distribution. Both axon guidance molecules have unexpected angiogenic effects,^109^ while perivascular cells, such as osteoclasts, regulate OA pain manifestation through axon guidance factor expression.

Sensory nerves sprout across bone fracture sites during normal healing,^110^ which indicates a self-protection mechanism to prevent untimely weight bearing by injured limbs. Abnormal innervation occurs during all stages of the pathology of OA and is the main cause of continuously aggravated osteoarthritis pain. At an early stage, BMLs can be detected on MRI and occur along with the upregulation of neurogenesis-associated genes,^10^ such as *G protein-coupled receptor 158* (*Gpr 158*), which has a neurotransmitter binding function, and ATPase H^+^-transporting lysosomal protein (ATP6V0D2), which plays a role in not only neuron projection but also osteoclast-osteoblast interactions.^111,112^ Neovascular invasion along nerve fibers breaching the tidemark has been observed in clinical samples, while similar pathological changes are present in osteophytes in knee OA.^113^ Calcitonin gene-related peptide-immunopositive (CGRP-IP) sensory nerve innervation has been shown to be significantly associated with OA pain manifestation, with the percentage of subchondral bone plate cavities containing CGRP-IP sensory nerves being obviously higher in the human symptomatic OA group than in the asymptomatic group.^114^

Taken together, these findings indicate that the microenvironment of subchondral bone in OA could be characterized by an elevated bone turnover rate, abnormal microstructures, overactive TGF-β, hypervascularization and aberrant sensory nerve innervation. These pathologies interact with each other and facilitate OA progression separately or in concert.

## Subchondral bone microenvironment and cartilage degeneration

### Coupling of angiogenesis and osteogenesis contributes to cartilage erosion

Accumulating evidence suggests that bottom-up vascularization from subchondral bone plays a larger role than top-down vessel invasion originating from synovial tissue or synovium during cartilage erosion in OA. Matrix-digesting proteases, such as MMPs, act as “road sweepers” for the neovasculature, and inhibitors purified from cartilage called tissue inhibitors of metalloproteinases (TIMPs) are believed to help maintain the avascular status.^115^ In the superficial cartilage layer, TIMP-1-positive chondrocytes have been found to be more abundant in OA samples than in the negative control, but in the deep zone near the bony plate, there is no significant difference.^103^ This means that vascular resistance in the superficial layer of cartilage remains better than that in the deep layer in OA, suggesting a pivotal role of subchondral angiogenesis during cartilage destruction.

Endothelial cells erode the cartilage matrix during bone elongation, thus creating space for substantial osteogenesis.^19,95^ Specifically, endothelial cells present 40-fold increased MMP-9 expression compared with osteoclasts during entochondrostosis, and the conditional knockout of MMP-9 in the postnatal endothelium leads to osteogenic defects and abnormally large growth plates, suggesting a critical role of endothelial-derived MMP-9 in cartilage resorption. In addition, the intra-articular administration of VEGF can accelerate OA progression in rodent models,^116^ while anti-VEGF treatment can ameliorate cartilage degradation.^117^ These data further indicate the cartilage resorption effect of neovascularization in both osteoarthritis and endochondral ossification.

Important research performed by Xie et al. has shown the critical role of preosteoclast-derived PDGF-BB in type H vessel genesis and bone remodeling.^118^ The bone turnover rate is accelerated in the subchondral bone in OA, especially in BML zones;^47^ the overactivation of osteoclastogenesis generates high levels of angiogenic factors, such as PDGF-BB and VEGF, and in turn, CD31^+^Endomucin^+^ type H vessels accelerate the turnover rate and aggravate OA progression.^119^ Inflammatory factors in the joint space, such as TNF-α, alter subchondral bone angiogenesis and MSC migration by inducing the expression of a newly discovered pathological angiogenesis regulator, leucine-rich-alpha-2-glycoprotein 1 (LRG1).^120^ Consistent with the higher clinical incidence of OA in females, recent literature has reported a more pronounced change in serum markers of neovascularization in female OA patients than in male OA patients.^121^

### Abnormal mechanical support and cartilage metabolism

#### Mechanical stimulation and cartilage homeostasis

Articular cartilage needs proper mechanical loading to remain healthy,^122^ and both overloading and underloading can be detrimental. Underloading, or even nonweight-bearing, leads to cartilage atrophy or degradation,^123,124^ while overloading, which is most often caused by obesity or deformity, results in cartilage fissures,^64^ bone sclerosis and osteoarthritis.^125,126^ A recent multicenter data analysis has shown that static loading, mainly from body weight, and cumulative loading, such as that from excessive daily walking, are associated with the worsening of cartilage damage, especially in the medial tibiofemoral joint.^127^ In the acute stage of cartilage impact experiments, mitochondrial dysfunction presents as an early response, which is followed by chondrocyte death, but chondrocytes from weight-bearing areas are more resistant to impact-induced cell death.^128^ According to Song et al., the gremlin-1-NF-κB signaling pathway plays an essential role in excessive load-induced cartilage degeneration.^129^ Proper biomechanical stimulation not only facilitates matrix synthesis by chondrocytes, including matrix proteins, collagen and glycosaminoglycan,^130^ but also helps maintain hydraulic permeability for necessary substance exchange.^131,132^ Instead of physiological stimulation, excessive mechanical loading upregulates p38 phosphorylation and MMP-13 expression,^133^ thus inducing matrix degradation and proteoglycan loss.^134^ Moreover, subchondral bone perforations containing neovasculature and nerve fibers are colocalized with mechanical stress points,^135^ indicating that biomechanical signals influence not only cartilage metabolism but also subchondral bone plate remodeling.

#### MSC biological behavior under biomechanical signals

In vitro, MSCs can respond to mechanical stimulation and differentiate toward various lineages without altering the culture medium.^136^ Ex vivo experiments have shown that chemoattractant-induced MSC migration is restrained under the influence of extrinsic force, which inhibits autonomous tissue repair. Subsequently, in the advanced stage of injury, cell density loss and matrix erosion can be observed by histology.^128^ Under elevated cyclic compression in vivo, osteogenic differentiation increases as a result of various actions. MSCs under abnormal stress demonstrate an increased angiogenic capacity, and conditioned medium from mechanically stimulated MSCs results in the significant promotion of angiogenesis compared with that from the negative control.^137^ Multiple cytokines detected in the abovementioned medium are closely related to vascular formation, such as FGF, TGF-β and MMP-2. Other research has indicated that osteogenic differentiation is promoted by BMP-dependent signaling because it can be reversed by rhNoggin administration.^138^ Therefore, MSCs in sclerotic subchondral bone could exhibit an excessive angiogenic ability, which facilitates cartilage degeneration, as mentioned above.

### OA subchondral microenvironment and chondrocyte senescence

Senescent phenotypical features are normally observed in chondrocytes in OA, such as upregulated β-galactosidase (β-gal) activity, upregulated cell cycle inhibitor p16^INK4a^ expression, shortened telomeres and SASPs.^139^ Interestingly, the transplantation of aging chondrocytes into a healthy knee joint leads to the OA phenotype, including severe cartilage destruction,^140^ which indicates an underlying role of chondrocyte senescence in the onset and progression of OA. In addition, epigenetic alterations have been revealed in aged human OA samples.^141^ Chondrocyte senescence is closely related to the diseased microenvironment in subchondral bone. p16^INK4a^-positive senescent MSCs have been identified in subchondral bone trabeculae in an anterior cruciate ligament transection (ACLT)-induced OA model, as well as in an aging mouse model (19–20 months old)^142^ and a spontaneous OA mouse model.^143^ Senescence-associated phenotypical features of MSCs are observed in subchondral bone in OA, including G0/G1-phase stagnation, increased β-gal expression, and decreased S-phase entry.^144^

Given the presence of senescent chondrocytes and subchondral pathologies in OA, we further investigated the intrinsic association between the subchondral microenvironment and cartilage senescence. SASPs derived from senescent or hypertrophic chondrocytes recruit immature osteoclasts and facilitate maturation. Moreover, exosomal microRNA from osteoclasts promotes hypertrophic transformation via Smad2 inhibition.^145^ In addition, senescent MSCs derived from synovium and subchondral bone in OA have been proven to be detrimental to cartilage homeostasis, both in vivo and ex vivo. Overall, senescence can be identified as a global pathological characteristic that affects all participants in the OA microenvironment. SASPs from multiple sources affect proliferation, differentiation and other cell functions. However, further studies are required to elucidate the underlying mechanism.

## Subchondral bone microenvironment and OA pain

### Mechanism of OA pain

**Fig. 5:**
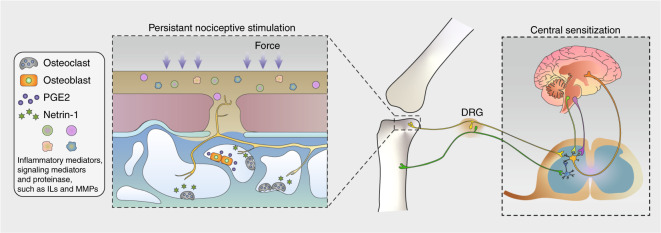
Schematic diagram of OA pain. Osteoclast-derived netrin-1 guides sensory nerve innervation in the osteochondral area, and multiple signaling mediators give rise to persistent nociceptive stimulation. The continuous input of pain signals causes central sensitization, suppresses the descending inhibitory system, and brings sympathetic referred pain away from the area of the arthritis lesion

It is a common complaint of orthopedists that osteoarthritis pain is not associated with the radiographic presentation (Fig. 5). Recent work has shown a relatively weak relationship between cartilage loss and OA joint pain. During a 36-month clinical survey, a loss of cartilage thickness of 0.01 mm over 2 years was responsible for an increase of 0.32 in the WOMAC score,^146^ indicating that the deep pathogenesis of OA pain is unclear and that further consideration might be necessary when dealing with worsening OA pain. Rather than cartilage, the abovementioned CGRP-IP nerve fibers in the osteochondral plate channels have been found to be more critical to OA pain in both humans and rats.^114^

Pain is often divided into three major types: nociceptive pain, which is caused by tissue damage in situ; neuropathic pain, which is caused by sensory nerve lesions; and idiopathic pain, whose etiology cannot be clearly explained.^147^ The classification of pain in OA patients has long been neglected even though NSAID administration does not work for all patients complaining of stubborn joint discomfort.^148^ In fact, neuropathic pain is more likely to react to nonstandard analgesics, such as tricyclic antidepressants. Recently, there has been growing evidence of the neuropathic component of OA pain,^149^ and a deeper understanding of the pain mechanism could help in dealing with persistent joint pain. In contrast to structural changes in bone, cartilage and synovium, pain is a subjective phenomenon; thus, psychological factors may be involved, which cannot be ignored.

### Nociceptive pain in situ

Around the knee joint, there are sensory and sympathetic nerve fibers that control nociception and proprioception.^150^ As articular cartilage functions without internal vessels and nerves, nociceptive pain is naturally related to peripheral tissues, including the subchondral bone, meniscus and synovium. Experimental OA pain can be eliminated by the selective targeting of nociceptive primary afferents,^151^ confirming the existence and therapeutic potential of nociceptive pain in osteoarthritis. Joint space inflammation plays a key role in OA chronic pain and acts as an initiator of cascade reactions. For example, synovial serine proteinase activates a group of G protein-coupled receptors called PARs and leads to several chronic pain conditions.^152^ Neuropeptide substance P (SP), a neurotransmitter in the mammalian central and peripheral nervous systems,^153^ has been found to be an injury-induced factor^154^ that plays a critical role in the pathogenesis of OA. As part of the neurokinin-1 (NK1) pain pathway, SP levels are elevated in both cerebrospinal^155^ and synovial fluid^156^ in OA patients with pain. The inhibition of SP-NK1 signaling with NK1 receptor antagonists results in partial anesis of nociceptive pain.^157^ Growth factors and cytokines also participate in OA pain via both cartilage degradation and nociceptive stimulation. Pain relief has been observed for therapies targeting inflammatory mediators such as TNF,^158^ IL-1 β,^159^ IL-6^160^ and PG-E2,^161^ signaling mediators such as p38,^162^ HIF^163^ and RUNX2,^164^ and proteinases such as MMP^165^ and ADAMTS-5.^166^

### Neuropathic pain and sensitization in subchondral bone in OA

A systematic review has shown a 23% incidence of neuropathic pain among knee or hip OA patients, with considerable heterogeneity,^167^ and gabapentin has been suggested to be helpful in dealing with OA pain.^168^ Nerve fiber lesions can be tested by the presence of positive and negative signs.^169^ Positive signs include hyperalgesia and allodynia, while negative signs include dysesthesia, numbness and a loss of vibration perception. The peripheral sensory nervous system transforms into a hypersensitive state, which can be activated by unproductive stimuli, and this supersensitive condition begins in the first few hours after injury or inflammation.^170^ Both in vivo electrophysiological^171^ examinations and subjective questionnaires for quantitative sensory testing^147^ have revealed peripheral sensitization in OA patients or animal models. While most peripheral nociceptors remain silent, inflammatory factors lower their thresholds in the OA environment,^172^ and nociceptive signals are transmitted through ascending pathways.

Constant nociceptive signals are transmitted by afferent neurons and are received by interneurons through synapses in the dorsal horn of the spinal cord, resulting in prolonged hyperexcitability^150^ or so-called central sensitization in OA. Quantitative sensory testing (QST) of the pressure pain threshold (PPT) has confirmed the existence of central sensitization among OA patients. A low PPT value in the OA area indicates peripheral sensitization, while central sensitization presents as abnormal PPT values in remote areas.^173^ Central sensitization directly leads to separation of the nociceptive activation site and the pain location, which happens to be an osteoarthritis characteristic.^174^ The precise mechanism of neurosensitization remains unclear, but successful total hip^175^ or knee^176^ arthroplasty can reverse this sensory abnormality, which indicates that periarticular injury or stimulation might be responsible for central nervous system hypersensitivity. In addition, descending inhibitory pathways in OA patients are weakened by multiple elements. Descending neural signals are disrupted in OA animal models, and medications such as norepinephrine reuptake inhibitors or opioid analgesics might help relieve intense OA pain. Tapentadol, an analgesic molecule that acts via both mechanisms, has been proven to be helpful against neuropathic pain in knee OA.^177^

### Abnormal sensory nerve innervation links nociceptive and hypersensitive pain in OA

Elegant work has revealed aberrant nerve innervation and regulation in OA.^178^ One week after ACLT surgery, invasion of the nociceptive sensory nerve near the chondro-osseous region could be observed by CGRP immunostaining, while the number of TRAP^+^ cells and CGRP concentration were unaltered in the control group. This alteration, which was reflected by the number and density of nerve endings, was worse 8 weeks after surgery.

As previously described, the bone turnover rate and osteoclastogenesis are upregulated in subchondral bone, and the axon guidance molecule netrin-1, which is secreted by subchondral TRAP^+^ cells, promotes and guides sensory nerve axon growth through the DCC receptor. Analogous sensory innervation induced by osteoclasts and netrin-1 has been observed in porous endplates and mediates spinal hypersensitivity.^161^ This evidence explains the occurrence of pain relief after alendronate administration in certain areas in OA patients.

Overactive bone remodeling in subchondral bone is associated with bone sclerosis, angiogenesis, microstructural disruption and sensory innervation. In addition to the osteoclast-derived netrin-1-induced axon distribution, prostaglandin E2 (PGE2) secreted by osteoblasts upregulates Na_v_1.8, a voltage-gated sodium channel expressed in dorsal root ganglion neurons and fibers, thus inducing hypersensitivity in the peripheral nerve environment.^179^ Specifically, PGE2 and its receptor EP4 stimulate the phosphorylation of protein kinase A (PKA) and cAMP element-binding protein (CREB1), thus enhancing Na_v_1.8 transcription in dorsal root ganglion (DRG) neurons. Semaphorin 3A (Sema3A), derived from neurons, regulates bone remodeling in an osteoblast-independent manner,^180^ and peripheral sensory nerves stimulate bone formation through Wnt signaling.^181^ This evidence indicates the feedback regulation of sensory nerves in bone remodeling. In fact, PGE2 expression is significantly higher in areas of decreased bone density, such as in an osteoporotic mouse model. The peripheral sensory nerve detects the bone density by sensing the PGE2 concentration and stimulates the phosphorylation of CREB1 in the hypothalamus, thus maintaining the bone mass by regulating sympathetic nerve activity.^182^ This evidence reveals the elaborate feedback loop involving subchondral bone remodeling, nerve innervation and hypersensitivity, which could be potential targets of future interventions for OA.

## Targeting of the subchondral bone microenvironment for OA and pain

Given the heterogeneity of symptoms and reactions to medications among OA patients, personalized treatment should be administered after complete assessment of the pathology according to the guidelines for different situations. Regarding the timing and method of surgery for end-stage OA, we need to be aware that total or partial arthroplasty might not allow complete remission to be achieved in all patients.^183^ In addition to various surgeries, an etiological analysis and targeted therapies might improve the prognosis (Table 1).

**Table 1:** Promising pharmacological interventions targeting subchondral bone in OA

Intervention	Mechanism of action	Object	Ref.
Systemic administration			
Bisphosphonate	Decreases turnover rate in subchondral bone	A, H	^50,186,216^
TGF-β inhibitors	Inhibit TGF-induced abnormal remodeling	A	^184^
Osteoprotegerin	Modulates RANKL/OPG equilibrium and remodeling	A	^185^
VEGF antibody	Neutralizes VEGF and reduce angiogenesis	A	^104,192^
Angiogenesis inhibitors	Inhibit endothelial cell proliferation	A	^194^
NGF antibody	Inhibits NGF signaling and OA pain	H	^193,217^
mTOR inhibitors	Reduce SASP secretion and delay cellular senescence	A	^204^
Local delivery			
TGF-β inhibitors	Inhibit TGF-induced local abnormal remodeling	A	^25^
Angiogenesis inhibitors	Block endothelial cell proliferation and migration	A	^195^
CRISPR/Cas9 system	Local ablation of NGF	A	^218^

### Subchondral bone microenvironment regulation

#### Regulation of uncoupled bone remodeling

High-level bone remodeling has been proven to be responsible for mechanically induced OA, and the inhibition of osteoclast-induced bone resorption could significantly attenuate OA symptoms in a mouse model. TGF-β and osteoclast overactivation have been observed at the onset of OA, and OC-induced matrix resorption increases TGF-β release and activation. The local neutralization of TGF-β in subchondral tissue by knocking out its receptor on MSCs reverses aberrant bone remodeling and excessive angiogenesis, thus attenuating cartilage degradation in ACLT mouse models.^25^ In addition, the effective ingredient of a traditional Chinese medicine, halofuginone, has shown a therapeutic effect in OA patients after systemic delivery by downregulating TGF-β signaling, decreasing bone remodeling and reducing vascularization.^184^ Interestingly, the systemic administration of OPG presented a cartilage-protective effect in a medial meniscectomy model, while OPG did not affect chondrocytes ex vivo,^185^ indicating the OPG-induced inhibition of osteoclast-induced bone remodeling. Runx-2 overexpression-induced excessive bone resorption leads to instability in the hind limbs, and these effects can be rescued by pamidronate, a well-known bone remodeling inhibitor.^186^ The bone turnover rate of subchondral bone has been reported to be both increased and decreased in various studies. Recent ideas interpret these observations as representing different temporal stages of OA progression, which indicates that there is a relatively low remodeling rate and a reduced level of osteopenia in the early stage that initiate a rapid remodeling process and cause subsequent OA pathologies. Otherwise, novel biomaterials or medications precisely targeting osteoclasts^187–189^ and bone remodeling^190,191^ have great potential in local interventions for subchondral bone. In conclusion, the legitimate control of subchondral bone remodeling at appropriate times and locations holds great therapeutic potential.

#### Inhibition of aberrant angiogenesis

A positive feedback loop has been identified in bone-vascular-cartilage crosstalk during OA involving chondrocytes, mTORC-1, VEGF-A and type H vessels, and the inhibition of angiogenesis by bevacizumab, a VEGF-A antibody, can terminate this feedback loop and thus rescue OA progression.^104,192,193^ Blocking VEGF-induced angiogenesis has shown a significant suppressive effect on vascular invasion and cartilage destruction. Other antiangiogenic factors, such as PPI-2548,^194^ TSP-1,^195^ Chm-1^196^ and angiostatin,^197,198^ have presented similar cartilage protective effects in multiple studies. To make room for new tissue, the matrix of bone or cartilage must be eroded before vessel growth can occur. This process, which is mostly induced by MMP-2, MMP-7 and membrane type 1 matrix metalloproteinase (MT1-MMP),^199^ allows endothelial cells to migrate and further degrade the cartilage matrix, while the inhibition of MMP signaling decreases cellular migration and angiogenesis.^200^ In addition, MMP releases VEGF in multiple matrices and thus accelerates angiogenesis in various diseases, including tumor invasion.^201^ The extracellular domain of FGF receptor type 1 in the vascular basement membrane can be hydrolyzed by MMP-2 to block the intracellular signaling pathway,^202^ thus inhibiting vascularization. In addition to angiogenic factors, several antiangiogenic molecules are also released by MMPs, such as endostatin, arrestin, canstatin and tumstatin, which are derived from various types of collagen. Overall, while antiangiogenic factors have a great latent capacity for OA therapy, further research is needed to clarify the exact timing and methods for the use of MMPs and their inhibitors in treating OA.

#### Targeting senescence

The selective removal of senescent cells has become a hot spot of research in OA therapeutics. Using drugs to block the antiapoptotic ability of senescent cells and their ability to secrete proinflammatory factors has also been explored as a method to remove senescent cells. In addition to inflammatory factors and excessive wear, senescent cells isolated from the cartilage and synovial tissues of OA patients have been found to contribute to OA progression.^142^ In p16-3MR transgenic mice (an animal model of aging in which p16lnk4a-positive senescent cells can be monitored), researchers injected ganciclovir (GCV) to induce apoptosis and selectively remove senescent cells. In addition, they tested a recently discovered senolytic (UBX0101) that could selectively remove senescent cells, and the results showed a clearing effect on senescent cells. In addition, the senescence of chondrogenic progenitor cells (CPCs) hinders the formation of chondrocytes, and senescent CPCs show an increase in IL-1β synthesis. In an intermittent hydrostatic pressure (IHP) mouse model, the senolytics dasatinib and quercetin were used to selectively induce the apoptosis of aging CPCs in vivo and in vitro, which might promote cartilage formation.^203^ Oxidative stress is one of the most important reasons for the formation of senescent cells, so reducing oxidative stress may be a way to eliminate senescent MSCs. Although the mechanism of cellular senescence in OA is not completely clear, the selective removal of senescent cells, especially in subchondral bone in joints, attenuates the development of posttraumatic OA, which provides new insights into treatments targeting senescent cells in OA.^142^

At present, there are also cytokine-neutralizing antibody therapies for use against senescent cells. Rapamycin is an mTOR inhibitor, and treatment with rapamycin could reduce the expression of MMP-3, MMP-13, IL-1 and IL-6 in rabbit annulus fibrosus stem cells (AFSCs), inhibit mTOR signaling, reduce SASP secretion, regulate the inflammatory response in vivo, and inhibit cell senescence, thus showing potential for the treatment of age-related diseases,^204^ including OA.

### Chronic pain management

Since OA pain is caused by multiple pathogenic processes, including nociceptive and neuropathic mechanisms, and appears to be heterogeneous, patients of different subtypes should be treated differently.^205^ Large numbers of pain-relieving drugs or bioactive constituents have been reported in recent years, but there is no single medication that works for all subtypes of OA based on present clinical experience.

NSAIDs are recommended as first-line agents for knee OA pain in the AAOS guidelines (2nd edition).^206^ Traditional nonselective COX inhibitors, such as indomethacin and ibuprofen, have substantial side effects in the alimentary canal. Selective COX-2 inhibitors, such as celecoxib and etoricoxib, present a significantly lower risk of gastrointestinal events without decreasing cardiovascular safety.^207,208^ COX-2 acts as a rate-limiting enzyme in osteoblasts, and the inhibition of COX-2 or PGE2-EP4 signaling in subchondral bone results in a tendency toward adipogenesis during MSC differentiation, thus decreasing osteogenesis and angiogenesis and alleviating OA pain. Nonopioid central analgesics, such as tramadol, have been applied in OA pain management for years. The additional administration of tramadol was recommended for senile OA patients who were refractory to NSAIDs in a clinical retrospective study.^209^ In addition, some opioid medications, such as transdermal buprenorphine, have been applied in clinical practice and have achieved good results.^210,211^

The intraarticular injection of corticosteroids for the treatment of OA pain seems to be common in clinical practice and is aimed at reducing inflammation inside the joint space, but it has been widely questioned in recent years. Research by Bruno et al.^212^ has suggested that intraarticular corticosteroids are associated with a moderate improvement in pain, but this is based on low-quality evidence. When compared with the injection of saline, the local injection of triamcinolone for 2 years resulted in significantly greater cartilage loss and no improvement in pain management.^213^ Patients who received physical therapy exhibited a painless and well-functioning joint, unlike patients who received intraarticular glucocorticoid injections.^214^ In addition, intra-articular hyaluronic acid therapy is not supported or discouraged in the AAOS guidelines^206^ or a report by Grace et al.^215^

## Conclusion and perspective

Complicated anatomical structures and interactive pathologies increase the difficulty of the clinical diagnosis and pharmaceutical treatment of OA. The intricate regulatory mechanisms between cartilage and subchondral bone include both mechanical and biological factors and play critical roles in the onset and progression of OA. Subchondral bone lies subjacent to the cartilage layer and provides mechanical and nutritional support for the cartilage. Multiple studies have reported that asymptomatic subchondral bone marrow lesions present earlier than OA pain or cartilage destruction. Recent research elucidating bone-cartilage crosstalk and subchondral bone metabolism provides a better understanding of this complex of whole-joint disorders. In this review, we summarized the recent progress in research on OA with a focus on subchondral bone. Briefly, abnormal mechanical loading leads to increased subchondral bone turnover, while angiogenesis and nerve innervation facilitate chondrocyte hypertrophy and cartilage degradation. These pathologies in turn accelerate bone remodeling beneath the tidemark, and finally, aberrant mechanical support due to subchondral bone sclerosis induces cartilage destruction. In conclusion, the inefficiency of therapies for local cartilage repair or inflammation inhibition (including arthroscopic debridement and injections), along with the promise of methods for regulating the subchondral microenvironment, suggests a new orientation for OA management in the future.

## References

[1] Hunter DJ, Bierma-Zeinstra S (2019). Osteoarthritis. Lancet.

[2] Wilson AJ, Murphy WA, Hardy DC, Totty WG (1988). Transient osteoporosis: transient bone marrow edema?. Radiology.

[3] Zanetti M, Bruder E, Romero J, Hodler J (2000). Bone marrow edema pattern in osteoarthritic knees: correlation between MR imaging and histologic findings. Radiology.

[4] Roemer FW (2009). MRI-detected subchondral bone marrow signal alterations of the knee joint: terminology, imaging appearance, relevance and radiological differential diagnosis. Osteoarthr. Cartil..

[5] Bowes MA (2016). Osteoarthritic bone marrow lesions almost exclusively colocate with denuded cartilage: a 3D study using data from the osteoarthritis initiative. Ann. Rheum. Dis..

[6] Guermazi A (2012). Prevalence of abnormalities in knees detected by MRI in Adults without knee osteoarthritis: population based observational study (Framingham Osteoarthritis Study). BMJ.

[7] Sowers MF (2003). Magnetic resonance-detected subchondral bone marrow and cartilage defect characteristics associated with pain and X-ray-defined knee osteoarthritis. Osteoarthr. Cartil..

[8] Felson DT (2001). The association of bone marrow lesions with pain in knee osteoarthritis. Ann. Intern. Med..

[9] Perry TA (2020). Association between bone marrow lesions & synovitis and symptoms in symptomatic knee osteoarthritis. Osteoarthr. Cartil..

[10] Kuttapitiya A (2017). Microarray analysis of bone marrow lesions in osteoarthritis demonstrates upregulation of genes implicated in osteochondral turnover, neurogenesis and inflammation. Ann. Rheum. Dis..

[11] Lowitz T (2013). Bone marrow lesions identified by MRI in knee osteoarthritis are associated with locally increased bone mineral density measured by QCT. Osteoarthr. Cartil..

[12] Raynauld JP (2008). Correlation between bone lesion changes and cartilage volume loss in patients with osteoarthritis of the knee as assessed by quantitative magnetic resonance imaging over a 24-month period. Ann. Rheum. Dis..

[13] Muratovic D (2019). Bone marrow lesions in knee osteoarthritis: regional differences in tibial subchondral bone microstructure and their association with cartilage degeneration. Osteoarthr. Cartil..

[14] Lee CH (2010). Regeneration of the articular surface of the rabbit synovial joint by cell homing: a proof of concept study. Lancet.

[15] Shimomura K (2010). The influence of skeletal maturity on allogenic synovial mesenchymal stem cell-based repair of cartilage in a large animal model. Biomaterials.

[16] Zhong J (2016). Crosstalk between adipose-derived stem cells and chondrocytes: when growth factors matter. Bone Res..

[17] McGonagle D, Baboolal TG, Jones E (2017). Native joint-resident mesenchymal stem cells for cartilage repair in osteoarthritis. Nat. Rev. Rheumatol..

[18] Zhang W (2013). The use of type 1 collagen scaffold containing stromal cell-derived factor-1 to create a matrix environment conducive to partial-thickness cartilage defects repair. Biomaterials.

[19] Romeo SG (2019). Endothelial proteolytic activity and interaction with non-resorbing osteoclasts mediate bone elongation. Nat. Cell Biol..

[20] Havelka S, Horn V, Spohrova D, Valouch P (1984). The calcified-noncalcified cartilage interface: the tidemark. Acta Biol. Hung..

[21] Silvast TS, Jurvelin JS, Lammi MJ, Toyras J (2009). PQCT study on diffusion and equilibrium distribution of iodinated anionic contrast agent in human articular cartilage–associations to matrix composition and integrity. Osteoarthr. Cartil..

[22] Flynn, C., M. Hurtig, C. & Linden, A. Z. Anionic contrast-enhanced microCT imaging correlates with biochemical and histological evaluations of osteoarthritic articular cartilage. *Cartilage*10.1177/1947603520924748 (2020). Online ahead of print. 10.1177/1947603520924748 PMC8804789 32456450

[23] Bhattarai, A. et al. Effects of human articular cartilage constituents on simultaneous diffusion of cationic and non-ionic contrast agents. *J Orthop Res*. 10.1002/jor.24824 (2020). Online ahead of print. 10.1002/jor.24824 PMC8048551 32767676

[24] Pan J (2009). In situ measurement of transport between subchondral bone and articular cartilage. J. Orthop. Res..

[25] Zhen G (2013). Inhibition of TGF-beta signaling in mesenchymal stem cells of subchondral bone attenuates osteoarthritis. Nat. Med..

[26] Kwan TS (2009). Modulation of OPG, RANK and RANKL by human chondrocytes and their implication during osteoarthritis. Rheumatol..

[27] Wang B, Jin H, Shu B, Mira RR, Chen D (2015). Chondrocytes-specific expression of osteoprotegerin modulates osteoclast formation in metaphyseal bone. Sci. Rep..

[28] Xiong J (2011). Matrix-embedded cells control osteoclast formation. Nat. Med..

[29] Wang B (2014). Chondrocyte beta-catenin signaling regulates postnatal bone remodeling through modulation of osteoclast formation in a murine model. Arthritis Rheumatol..

[30] Loeser RF (2013). Aging processes and the development of osteoarthritis. Curr. Opin. Rheumatol..

[31] Hu K, Olsen BR (2016). Osteoblast-derived VEGF regulates osteoblast differentiation and bone formation during bone repair. J. Clin. Invest..

[32] Imhof H (2000). Subchondral bone and cartilage disease: a rediscovered functional unit. Invest Radiol..

[33] Lyons TJ, McClure SF, Stoddart RW, McClure J (2006). The normal human chondro-osseous junctional region: evidence for contact of uncalcified cartilage with subchondral bone and marrow spaces. BMC Musculoskelet. Disord..

[34] Wu L, Prins HJ, Helder MN, C.A. vanBlitterswijk, Karperien M (2012). Trophic effects of mesenchymal stem cells in chondrocyte co-cultures are independent of culture conditions and cell sources. Tissue Eng. Part A..

[35] Wu L (2011). Trophic effects of mesenchymal stem cells increase chondrocyte proliferation and matrix formation. Tissue Eng. Part A..

[36] Caplan AI (2007). Adult mesenchymal stem cells for tissue engineering versus regenerative medicine. J. Cell Physiol..

[37] Zhang S (2018). MSC exosomes mediate cartilage repair by enhancing proliferation, attenuating apoptosis and modulating immune reactivity. Biomaterials.

[38] Wong KL (2020). Intra-articular injections of mesenchymal stem cell exosomes and hyaluronic acid improve structural and mechanical properties of repaired cartilage in a rabbit model. Arthroscopy.

[39] Wang R (2020). Intra-articular delivery of extracellular vesicles secreted by chondrogenic progenitor cells from mrl/mpj superhealer mice enhances articular cartilage repair in a mouse injury model. Stem Cell Res. Ther..

[40] Liu C (2020). Kartogenin enhances the therapeutic effect of bone marrow mesenchymal stem cells derived exosomes in cartilage repair. Nanomedicine.

[41] Sanchez C (2005). Subchondral bone osteoblasts induce phenotypic changes in human osteoarthritic chondrocytes. Osteoarthr. Cartil..

[42] Chen X, Zhi X, Wang J, Su J (2018). RANKL signaling in bone marrow mesenchymal stem cells negatively regulates osteoblastic bone formation. Bone Res..

[43] Nakashima T (2011). Evidence for osteocyte regulation of bone homeostasis through RANKL expression. Nat. Med..

[44] Huiskes R, Ruimerman R, van Lenthe GH, Janssen JD (2000). Effects of mechanical forces on maintenance and adaptation of form in trabecular bone. Nature.

[45] Martinez-Calatrava MJ (2012). RANKL synthesized by articular chondrocytes contributes to juxta-articular bone loss in chronic arthritis. Arthritis Res. Ther..

[46] Xiong J (2018). Soluble RANKL contributes to osteoclast formation in adult mice but not ovariectomy-induced bone loss. Nat. Commun..

[47] Shabestari M, Vik J, Reseland JE, Eriksen EF (2016). Bone marrow lesions in hip osteoarthritis are characterized by increased bone turnover and enhanced angiogenesis. Osteoarthr. Cartil..

[48] Maas O, Joseph GB, Sommer G, Wild D, Kretzschmar M (2015). Association between cartilage degeneration and subchondral bone remodeling in patients with knee osteoarthritis comparing MRI and (99m)Tc-DPD-SPECT/CT. Osteoarthr. Cartil..

[49] Wang F (2019). The bone marrow edema links to an osteoclastic environment and precedes synovitis during the development of collagen induced arthritis. Front Immunol..

[50] Siebelt M (2014). Inhibited osteoclastic bone resorption through alendronate treatment in rats reduces severe osteoarthritis progression. Bone.

[51] Mohan G (2013). Pre-emptive, early, and delayed alendronate treatment in a rat model of knee osteoarthritis: effect on subchondral trabecular bone microarchitecture and cartilage degradation of the tibia, bone/cartilage turnover, and joint discomfort. Osteoarthr. Cartil..

[52] Burr DB, Gallant MA (2012). Bone remodelling in osteoarthritis. Nat. Rev. Rheumatol..

[53] Frost HM (2001). From Wolff’s law to the utah paradigm: insights about bone physiology and its clinical applications. Anat. Rec..

[54] Gatenholm B, Lindahl C, Brittberg M, Stadelmann VA (2019). Spatially matching morphometric assessment of cartilage and subchondral bone in osteoarthritic human knee joint with micro-computed tomography. Bone.

[55] Holzer LA (2020). Microstructural analysis of subchondral bone in knee osteoarthritis. Osteoporos. Int..

[56] Pouran B (2017). Solute transport at the interface of cartilage and subchondral bone plate: effect of micro-architecture. J. Biomech..

[57] Bonewald LF (2011). The amazing osteocyte. J. Bone Min. Res..

[58] Tang SY, Herber RP, Ho SP, Alliston T (2012). Matrix metalloproteinase-13 is required for osteocytic perilacunar remodeling and maintains bone fracture resistance. J. Bone Min. Res..

[59] Qing H (2012). Demonstration of osteocytic perilacunar/canalicular remodeling in mice during lactation. J. Bone Min. Res..

[60] Kogawa M (2013). Sclerostin regulates release of bone mineral by osteocytes by induction of carbonic anhydrase 2. J. Bone Min. Res..

[61] Mazur CM (2019). Osteocyte dysfunction promotes osteoarthritis through MMP13-dependent suppression of subchondral bone homeostasis. Bone Res..

[62] Chen Y (2018). Subchondral trabecular rod loss and plate thickening in the development of osteoarthritis. J. Bone Min. Res..

[63] Shiraishi K (2020). In vivo analysis of subchondral trabecular bone in patients with osteoarthritis of the knee using second-generation high-resolution peripheral quantitative computed tomography (HR-pQCT). Bone.

[64] Chen L (2020). Horizontal fissuring at the osteochondral interface: a novel and unique pathological feature in patients with obesity-related osteoarthritis. Ann. Rheum. Dis..

[65] Hoechel S, Deyhle H, Toranelli M, Muller-Gerbl M (2017). Osteoarthritis alters the patellar bones subchondral trabecular architecture. J. Orthop. Res..

[66] Schett G (2013). Diabetes is an independent predictor for severe osteoarthritis: results from a longitudinal cohort study. Diabetes Care..

[67] Chen Y (2017). Abnormal subchondral bone remodeling and its association with articular cartilage degradation in knees of type 2 diabetes patients. Bone Res..

[68] Xu X (2018). Transforming growth factor-beta in stem cells and tissue homeostasis. Bone Res..

[69] Annes JP, Munger JS, Rifkin DB (2003). Making sense of latent TGFbeta activation. J. Cell Sci..

[70] Yu Q, Stamenkovic I (2000). Cell surface-localized matrix metalloproteinase-9 proteolytically activates tgf-beta and promotes tumor invasion and angiogenesis. Genes Dev..

[71] Werb Z (1997). ECM and cell surface proteolysis: regulating cellular ecology. Cell.

[72] Agah A, Kyriakides TR, Lawler J, Bornstein P (2002). The lack of thrombospondin-1 (TSP1) dictates the course of wound healing in double-TSP1/TSP2-null mice. Am. J. Pathol..

[73] Lyons RM, Keski-Oja J, Moses HL (1988). Proteolytic activation of latent transforming growth factor-beta from fibroblast-conditioned medium. J. Cell Biol..

[74] Dickinson ME (1990). Chromosomal localization of seven members of the murine TGF-beta superfamily suggests close linkage to several morphogenetic mutant loci. Genomics.

[75] Teitelbaum SL (2000). Bone resorption by osteoclasts. Science.

[76] Ten DP, Hill CS (2004). New insights into TGF-beta-smad signalling. Trends Biochem Sci..

[77] Xian L (2012). Matrix IGF-1 maintains bone mass by activation of mTOR in mesenchymal stem cells. Nat. Med..

[78] Miyoshi H, Ajima R, Luo CT, Yamaguchi TP, Stappenbeck TS (2012). Wnt5a potentiates TGF-beta signaling to promote colonic crypt regeneration after tissue injury. Science.

[79] Mesa KR (2015). Niche-induced cell death and epithelial phagocytosis regulate hair follicle stem cell pool. Nature.

[80] Lovisa S (2015). Epithelial-to-mesenchymal transition induces cell cycle arrest and parenchymal damage in renal fibrosis. Nat. Med..

[81] Dole NS (2017). Osteocyte-intrinsic TGF-beta signaling regulates bone quality through perilacunar/canalicular remodeling. Cell Rep..

[82] Wang X (2018). Aberrant TGF-beta activation in bone tendon insertion induces enthesopathy-like disease. J. Clin. Invest..

[83] Janssens K (2000). Mutations in the gene encoding the latency-associated peptide of TGF-beta 1 cause Camurati-Engelmann disease. Nat. Genet..

[84] Whyte MP (2011). Camurati-Engelmann disease: unique variant featuring a novel mutation in TGFbeta1 encoding transforming growth factor beta 1 and a missense change in TNFSF11 encoding RANK ligand. J. Bone Min. Res..

[85] Buscemi L (2011). The single-molecule mechanics of the latent TGF-beta1 complex. Curr. Biol..

[86] Kusumbe AP, Ramasamy SK, Adams RH (2014). Coupling of angiogenesis and osteogenesis by a specific vessel subtype in bone. Nature.

[87] Hu Y (2020). Defactinib attenuates osteoarthritis by inhibiting positive feedback loop between H-type vessels and MSCs in subchondral bone. J. Orthop. Transl..

[88] Carmeliet P, Jain RK (2011). Molecular mechanisms and clinical applications of angiogenesis. Nature.

[89] Chen WC (2013). Cellular kinetics of perivascular MSC precursors. Stem Cells Int..

[90] Liu T (2018). PDGF-mediated mesenchymal transformation renders endothelial resistance to anti-VEGF treatment in glioblastoma. Nat. Commun..

[91] Sacchetti B (2007). Self-renewing osteoprogenitors in bone marrow sinusoids can organize a hematopoietic microenvironment. Cell.

[92] Ramasamy SK, Kusumbe AP, Wang L, Adams RH (2014). Endothelial notch activity promotes angiogenesis and osteogenesis in bone. Nature.

[93] Lechertier T (2020). Pericyte FAK negatively regulates Gas6/Axl signalling to suppress tumour angiogenesis and tumour growth. Nat. Commun..

[94] Bragdon B (2017). Earliest phases of chondrogenesis are dependent upon angiogenesis during ectopic bone formation in mice. Bone.

[95] Sun P (2020). Regulation of body length and bone mass by Gpr126/Adgrg6. Sci. Adv..

[96] Gerber HP (1999). VEGF couples hypertrophic cartilage remodeling, ossification and angiogenesis during endochondral bone formation. Nat. Med..

[97] Kubo S (2009). Blocking vascular endothelial growth factor with soluble Flt-1 Improves the chondrogenic potential of mouse skeletal muscle-derived stem cells. Arthritis Rheum..

[98] Matsumoto T (2009). Cartilage repair in a rat model of osteoarthritis through intraarticular transplantation of muscle-derived stem cells expressing bone morphogenetic protein 4 and soluble flt-1. Arthritis Rheum..

[99] Shukunami C, Hiraki Y (2001). Role of cartilage-derived anti-angiogenic factor, chondromodulin-i, during endochondral bone formation. Osteoarthr. Cartil..

[100] Pfander D, Cramer T, Deuerling D, Weseloh G, Swoboda B (2000). Expression of thrombospondin-1 and its receptor CD36 in human osteoarthritic cartilage. Ann. Rheum. Dis..

[101] Moses MA (1999). Troponin I is present in human cartilage and inhibits angiogenesis. Proc. Natl Acad. Sci. USA.

[102] Huang Y (2020). 3D high-frequency ultrasound imaging of cartilage-bone interface compared with micro-CT. Biomed. Res Int..

[103] Franses RE, McWilliams DF, Mapp PI, Walsh DA (2010). Osteochondral angiogenesis and increased protease inhibitor expression in OA. Osteoarthr. Cartil..

[104] Lu J (2018). Positive-feedback regulation of subchondral H-type vessel formation by chondrocyte promotes osteoarthritis development in mice. J. Bone Min. Res..

[105] Mapp PI, Walsh DA (2012). Mechanisms and targets of angiogenesis and nerve growth in osteoarthritis. Nat. Rev. Rheumatol..

[106] Walsh DA (1996). Innervation and neurokinin receptors during angiogenesis in the rat sponge granuloma. Histochem J..

[107] Walsh DA (2010). Angiogenesis and nerve growth factor at the osteochondral junction in rheumatoid arthritis and osteoarthritis. Rheumatology.

[108] Yu X (2019). NGF increases FGF2 expression and promotes endothelial cell migration and tube formation through PI3K/Akt and ERK/MAPK pathways in human chondrocytes. Osteoarthr. Cartil..

[109] Carmeliet P, Tessier-Lavigne M (2005). Common mechanisms of nerve and blood vessel wiring. Nature.

[110] Hukkanen M (1993). Rapid proliferation of calcitonin gene-related peptide-immunoreactive nerves during healing of rat tibial fracture suggests neural involvement in bone growth and remodelling. Neuroscience.

[111] Chen X (2018). Osteoblast-osteoclast interactions. Connect Tissue Res..

[112] Kim T (2010). ATP6v0d2 deficiency increases bone mass, but does not influence ovariectomy-induced bone loss. Biochem Biophys. Res Commun..

[113] Suri S (2007). Neurovascular invasion at the osteochondral junction and in osteophytes in osteoarthritis. Ann. Rheum. Dis..

[114] Aso K (2020). Contribution of nerves within osteochondral channels to osteoarthritis knee pain in humans and rats. Osteoarthr. Cartil..

[115] Moses MA, Sudhalter J, Langer R (1990). Identification of an inhibitor of neovascularization from cartilage. Science.

[116] Hamilton JL (2016). Targeting VEGF and its receptors for the treatment of osteoarthritis and associated pain. J. Bone Min. Res..

[117] Barranco C (2014). Osteoarthritis: animal data show VEGF blocker inhibits post-traumatic OA. Nat. Rev. Rheumatol..

[118] Xie H (2014). PDGF-BB secreted by preosteoclasts induces angiogenesis during coupling with osteogenesis. Nat. Med..

[119] Su W (2020). Angiogenesis stimulated by elevated PDGF-BB in subchondral bone contributes to osteoarthritis development. JCI Insight.

[120] Wang Y (2017). TNF-alpha-induced LRG1 promotes angiogenesis and mesenchymal stem cell migration in the subchondral bone during osteoarthritis. Cell Death Dis..

[121] Kisand K, Tamm AE, Lintrop M, Tamm AO (2018). New insights into the natural course of knee osteoarthritis: early regulation of cytokines and growth factors, with emphasis on sex-dependent angiogenesis and tissue remodeling. A pilot study. Osteoarthr. Cartil..

[122] Sun HB (2010). Mechanical loading, cartilage degradation, and arthritis. Ann. N. Y Acad. Sci..

[123] Hinterwimmer S (2004). Cartilage atrophy in the knees of patients after seven weeks of partial load bearing. Arthritis Rheum..

[124] Souza RB (2012). Effects of unloading on knee articular cartilage T1rho and T2 magnetic resonance imaging relaxation times: a case series. J. Orthop. Sports Phys. Ther..

[125] Messier SP (2013). Effects of intensive diet and exercise on knee joint loads, inflammation, and clinical outcomes among overweight and obese adults with knee osteoarthritis: the IDEA randomized clinical trial. JAMA.

[126] Kulkarni K, Karssiens T, Kumar V, Pandit H (2016). Obesity and osteoarthritis. Maturitas.

[127] Voinier D (2020). Using cumulative load to explain how body mass index and daily walking relate to worsening knee cartilage damage over two years: the MOST study. Arthritis Rheumatol..

[128] Delco ML, Bonnevie ED, Bonassar LJ, Fortier LA (2018). Mitochondrial dysfunction is an acute response of articular chondrocytes to mechanical injury. J. Orthop. Res..

[129] Chang SH (2019). Excessive mechanical loading promotes osteoarthritis through the gremlin-1-NF-κB pathway. Nat. Commun..

[130] Fahy N, Alini M, Stoddart MJ (2018). Mechanical stimulation of mesenchymal stem cells: implications for cartilage tissue engineering. J. Orthop. Res..

[131] Reynaud B, Quinn TM (2006). Anisotropic hydraulic permeability in compressed articular cartilage. J. Biomech..

[132] Hoenig E (2013). Mechanical properties of native and tissue-engineered cartilage depend on carrier permeability: a bioreactor study. Tissue Eng. Part A.

[133] Nakagawa K (2012). Cyclic compression-induced P38 activation and subsequent MMP13 expression requires Rho/ROCK activity in bovine cartilage explants. Inflamm. Res..

[134] Patwari P, Cheng DM, Cole AA, Kuettner KE, Grodzinsky AJ (2007). Analysis of the relationship between peak stress and proteoglycan loss following injurious compression of human post-mortem knee and ankle cartilage. Biomech. Model Mechanobiol..

[135] Iijima H (2016). Subchondral plate porosity colocalizes with the point of mechanical load during ambulation in a rat knee model of post-traumatic osteoarthritis. Osteoarthr. Cartil..

[136] Steinmetz NJ, Aisenbrey EA, Westbrook KK, Qi HJ, Bryant SJ (2015). Mechanical loading regulates human MSC differentiation in a multi-layer hydrogel for osteochondral tissue engineering. Acta Biomater..

[137] Kasper G (2007). Mesenchymal stem cells regulate angiogenesis according to their mechanical environment. Stem Cells.

[138] Schreivogel S, Kuchibhotla V, Knaus P, Duda GN, Petersen A (2019). Load-induced osteogenic differentiation of mesenchymal stromal cells is caused by mechano-regulated autocrine signaling. J. Tissue Eng. Regen. Med..

[139] Lopez-Otin C, Blasco MA, Partridge L, Serrano M, Kroemer G (2013). The hallmarks of aging. Cell.

[140] Diekman BO (2018). Expression of p16(INK) (4A) is a biomarker of chondrocyte aging but does not cause osteoarthritis. Aging Cell..

[141] Chen D (2017). Osteoarthritis: toward a comprehensive understanding of pathological mechanism. Bone Res..

[142] Jeon OH (2017). Local clearance of senescent cells attenuates the development of post-traumatic osteoarthritis and creates a pro-regenerative environment. Nat. Med..

[143] Malaise O (2019). Mesenchymal stem cell senescence alleviates their intrinsic and seno-suppressive paracrine properties contributing to osteoarthritis development. Aging (Albany NY)..

[144] Zhao Y (2017). Age-related changes in nucleus pulposus mesenchymal stem cells: an in vitro study in rats. Stem Cells Int..

[145] Dai, J. et al. Osteoclast-derived exosomal let-7a-5p targets Smad2 to promote the hypertrophic differentiation of chondrocytes. *Am. J. Physiol. Cell Physiol*. 10.1152/ajpcell.00039.2020 (2020). Online ahead of print. 10.1152/ajpcell.00039.2020 32374679

[146] Bacon K, LaValley MP, Jafarzadeh SR, Felson D (2020). Does cartilage loss cause pain in osteoarthritis and if so, how much?. Ann. Rheum. Dis..

[147] Thakur M, Dickenson AH, Baron R (2014). Osteoarthritis pain: nociceptive or neuropathic?. Nat. Rev. Rheumatol..

[148] Gregori D (2018). Association of pharmacological treatments with long-term pain control in patients with knee osteoarthritis: a systematic review and meta-analysis. JAMA.

[149] Dimitroulas T, Duarte RV, Behura A, Kitas GD, Raphael JH (2014). Neuropathic pain in osteoarthritis: a review of pathophysiological mechanisms and implications for treatment. Semin Arthritis Rheum..

[150] Malfait AM, Schnitzer TJ (2013). Towards a mechanism-based approach to pain management in osteoarthritis. Nat. Rev. Rheumatol..

[151] Iadarola MJ, Sapio MR, Raithel SJ, Mannes AJ, Brown DC (2018). Long-term pain relief in canine osteoarthritis by a single intra-articular injection of resiniferatoxin, a potent TRPV1 Agonist. Pain.

[152] McDougall, J. J. & Muley, M. M. The role of proteases in pain. In *Pain Control. Handbook of Experimental Pharmacology* Vol. 227 (ed. Schaible, H.G.) 239–260 (Springer, Berlin, Heidelberg, 2015). 10.1007/978-3-662-46450-2_12 25846622

[153] Mantyh PW, Pinnock RD, Downes CP, Goedert M, Hunt SP (1984). Correlation between inositol phospholipid hydrolysis and substance P receptors in rat CNS. Nature.

[154] Hong HS (2009). A new role of substance P as an injury-inducible messenger for mobilization of CD29^+^ stromal-like cells. Nat. Med..

[155] Lindh C, Liu Z, Lyrenas S, Ordeberg G, Nyberg F (1997). Elevated cerebrospinal fluid substance p-like immunoreactivity in patients with painful osteoarthritis, but not in patients with rhizopatic pain from a herniated lumbar disc. Scand. J. Rheumatol..

[156] Marshall KW, Chiu B, Inman RD (1990). Substance P and arthritis: analysis of plasma and synovial fluid levels. Arthritis Rheum..

[157] Warner SC (2017). Pain in knee osteoarthritis is associated with variation in the neurokinin 1/substance P receptor (TACR1) gene. Eur. J. Pain..

[158] Li H (2018). TNF-alpha increases the expression of inflammatory factors in synovial fibroblasts by inhibiting the PI3K/AKT pathway in a rat model of monosodium iodoacetate-induced osteoarthritis. Exp. Ther. Med..

[159] Fleischmann RM (2019). A phase ii trial of lutikizumab, an anti-interleukin-1alpha/beta dual variable domain immunoglobulin, in knee osteoarthritis patients with synovitis. Arthritis Rheumatol..

[160] Azim S (2018). Interleukin-6 and leptin levels are associated with preoperative pain severity in patients with osteoarthritis but not with acute pain after total knee arthroplasty. Knee.

[161] Ni S (2019). Sensory innervation in porous endplates by netrin-1 from osteoclasts mediates PGE2-induced spinal hypersensitivity in mice. Nat. Commun..

[162] Brown KK (2008). P38 MAP kinase inhibitors as potential therapeutics for the treatment of joint degeneration and pain associated with osteoarthritis. J. Inflamm. (Lond.)..

[163] Taheem DK, Jell G, Gentleman E (2020). Hypoxia inducible factor-1alpha in osteochondral tissue engineering. Tissue Eng. Part B Rev..

[164] Orfanidou T, Iliopoulos D, Malizos KN, Tsezou A (2009). Involvement of SOX-9 and FGF-23 in RUNX-2 regulation in osteoarthritic chondrocytes. J. Cell Mol. Med..

[165] Martel-Pelletier J (1984). Neutral proteases capable of proteoglycan digesting activity in osteoarthritic and normal human articular cartilage. Arthritis Rheum..

[166] Larkin J (2015). Translational development of an ADAMTS-5 antibody for osteoarthritis disease modification. Osteoarthr. Cartil..

[167] French HP, Smart KM, Doyle F (2017). Prevalence of neuropathic pain in knee or hip osteoarthritis: a systematic review and meta-analysis. Semin Arthritis Rheum..

[168] Wiffen PJ (2017). Gabapentin for chronic neuropathic pain in adults. Cochrane Database Syst. Rev..

[169] Finnerup NB (2016). Neuropathic pain: an updated grading system for research and clinical practice. Pain.

[170] Schaible HG (2009). Joint pain. Exp. Brain Res..

[171] McDougall JJ, Andruski B, Schuelert N, Hallgrimsson B, Matyas JR (2009). Unravelling the relationship between age, nociception and joint destruction in naturally occurring osteoarthritis of dunkin hartley guinea pigs. Pain.

[172] Liu-Bryan R, Terkeltaub R (2015). Emerging regulators of the inflammatory process in osteoarthritis. Nat. Rev. Rheumatol..

[173] Suokas AK (2012). Quantitative sensory testing in painful osteoarthritis: a systematic review and meta-analysis. Osteoarthr. Cartil..

[174] Woolf CJ (2011). Central sensitization: implications for the diagnosis and treatment of pain. Pain.

[175] Kosek E, Ordeberg G (2000). Abnormalities of somatosensory perception in patients with painful osteoarthritis normalize following successful treatment. Eur. J. Pain..

[176] Graven-Nielsen T, Wodehouse T, Langford RM, Arendt-Nielsen L, Kidd BL (2012). Normalization of widespread hyperesthesia and facilitated spatial summation of deep-tissue pain in knee osteoarthritis patients after knee replacement. Arthritis Rheum..

[177] Rinonapoli G, Coaccioli S, Panella L (2019). Tapentadol in the treatment of osteoarthritis: pharmacological rationale and clinical evidence. J. Pain. Res..

[178] Zhu S (2019). Subchondral bone osteoclasts induce sensory innervation and osteoarthritis pain. J. Clin. Invest..

[179] Zhu J (2020). Aberrant subchondral osteoblastic metabolism modifies NaV1.8 for osteoarthritis. Elife.

[180] Fukuda T (2013). Sema3A regulates bone-mass accrual through sensory innervations. Nature.

[181] Levi B (2017). “TrkA”cking why “no pain, no gain” is the rule for bone formation. Sci. Transl. Med..

[182] Chen H (2019). Prostaglandin E2 mediates sensory nerve regulation of bone homeostasis. Nat. Commun..

[183] Richmond J (2009). Treatment of osteoarthritis of the knee (Nonarthroplasty). J. Am. Acad. Orthop. Surg..

[184] Cui Z (2016). Halofuginone attenuates osteoarthritis by inhibition of TGF-beta activity and H-type vessel formation in subchondral bone. Ann. Rheum. Dis..

[185] Kadri A (2008). Osteoprotegerin inhibits cartilage degradation through an effect on trabecular bone in murine experimental osteoarthritis. Arthritis Rheum..

[186] Kadri A (2010). Inhibition of bone resorption blunts osteoarthritis in mice with high bone remodelling. Ann. Rheum. Dis..

[187] Wang L, Huang B, Chen X, Su J (2020). New insight into unexpected bone formation by denosumab. Drug Discov. Today.

[188] Wan L (2018). A magnetic-field guided interface coassembly approach to magnetic mesoporous silica nanochains for osteoclast-targeted inhibition and heterogeneous nanocatalysis. Adv. Mater..

[189] Yue Q (2017). Plasmolysis-inspired nanoengineering of functional yolk-shell microspheres with magnetic core and mesoporous silica shell. J. Am. Chem. Soc..

[190] Song H (2019). Reversal of osteoporotic activity by endothelial cell-secreted bone targeting and biocompatible exosomes. Nano Lett..

[191] Metavarayuth K (2019). Nanotopographical cues mediate osteogenesis of stem cells on virus substrates through BMP-2 intermediate. Nano Lett..

[192] Nagai T (2014). Bevacizumab, an anti-vascular endothelial growth factor antibody, inhibits osteoarthritis. Arthritis Res Ther..

[193] Schnitzer TJ (2019). Effect of tanezumab on joint pain, physical function, and patient global assessment of osteoarthritis among patients with osteoarthritis of the hip or knee: a randomized clinical trial. JAMA.

[194] Ashraf S, Mapp PI, Walsh DA (2011). Contributions of angiogenesis to inflammation, joint damage, and pain in a rat model of osteoarthritis. Arthritis Rheum..

[195] Hsieh JL (2010). Intraarticular gene transfer of thrombospondin-1 suppresses the disease progression of experimental osteoarthritis. J. Orthop. Res..

[196] Hayami T (2003). Expression of the cartilage derived anti-angiogenic factor chondromodulin-i decreases in the early stage of experimental osteoarthritis. J. Rheumatol..

[197] Kim YM (2002). Endostatin blocks vascular endothelial growth factor-mediated signaling via direct interaction with KDR/Flk-1. J. Biol. Chem..

[198] Kurosaka D (2003). Inhibition of arthritis by systemic administration of endostatin in passive murine collagen induced arthritis. Ann. Rheum. Dis..

[199] Bini A, Wu D, Schnuer J, Kudryk BJ (1999). Characterization of stromelysin 1 (MMP-3), matrilysin (MMP-7), and membrane type 1 matrix metalloproteinase (MT1-MMP) derived fibrin(Ogen) fragments D-dimer and D-like monomer: NH2-terminal sequences of late-stage digest fragments. Biochemistry.

[200] Webb AH (2017). Inhibition of MMP-2 and MMP-9 decreases cellular migration, and angiogenesis in in vitro models of retinoblastoma. BMC Cancer.

[201] Hawinkels LJ (2008). VEGF release by MMP-9 mediated heparan sulphate cleavage induces colorectal cancer angiogenesis. Eur. J. Cancer.

[202] Levi E (1996). Matrix metalloproteinase 2 releases active soluble ectodomain of fibroblast growth factor receptor 1. Proc. Natl Acad. Sci. USA.

[203] Dai H (2020). Eliminating senescent chondrogenic progenitor cells enhances chondrogenesis under intermittent hydrostatic pressure for the treatment of OA. Stem Cell Res. Ther..

[204] Gao C, Ning B, Sang C, Zhang Y (2018). Rapamycin prevents the intervertebral disc degeneration via inhibiting differentiation and senescence of annulus fibrosus cells. Aging..

[205] Yuan C (2020). Classification of four distinct osteoarthritis subtypes with a knee joint tissue transcriptome atlas. Bone Res..

[206] Jevsevar DS (2013). Treatment of osteoarthritis of the knee: evidence-based guideline, 2nd edition. J. Am. Acad. Orthop. Surg..

[207] Nissen SE (2016). Cardiovascular safety of celecoxib, naproxen, or ibuprofen for arthritis. N. Engl. J. Med..

[208] Chan FK (2010). Celecoxib versus omeprazole and diclofenac in patients with osteoarthritis and rheumatoid arthritis (CONDOR): a randomised trial. Lancet.

[209] Makris UE, Abrams RC, Gurland B, Reid MC (2014). Management of persistent pain in the older patient: a clinical review. JAMA.

[210] Gil HY (2019). A novel application of buprenorphine transdermal patch to relieve pain in the knee joint of knee osteoarthritis patients: a retrospective case-control study. J. Clin. Med..

[211] Da CB (2014). Oral or transdermal opioids for osteoarthritis of the knee or hip. Cochrane Database Syst. Rev..

[212] Da CB, Hari R, Juni P (2016). Intra-articular corticosteroids for osteoarthritis of the knee. JAMA.

[213] McAlindon TE (2017). Effect of intra-articular triamcinolone vs saline on knee cartilage volume and pain in patients with knee osteoarthritis: a randomized clinical trial. JAMA.

[214] Deyle GD (2020). Physical therapy versus glucocorticoid injection for osteoarthritis of the knee. N. Engl. J. Med..

[215] Lo GH, LaValley M, McAlindon T, Felson DT (2003). Intra-articular hyaluronic acid in treatment of knee osteoarthritis: a meta-analysis. JAMA.

[216] Laslett LL (2012). Zoledronic acid reduces knee pain and bone marrow lesions over 1 year: a randomised controlled trial. Ann. Rheum. Dis..

[217] Lane NE (2010). Tanezumab for the treatment of pain from osteoarthritis of the knee. N. Engl. J. Med..

[218] Zhao L (2019). Exploration of CRISPR/Cas9-based gene editing as therapy for osteoarthritis. Ann. Rheum. Dis..

